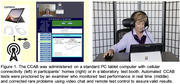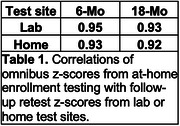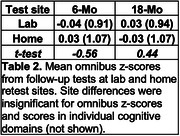# A Computerized Cognitive Assessment Battery Optimized for At‐Home Testing

**DOI:** 10.1002/alz.091520

**Published:** 2025-01-03

**Authors:** Kathleen Hall, Juliana Baldo, Peter Pebler, Timothy J Herron, Kristin Geraci, Michael Blank, Krista Schendel, Sandy J. Lwi, Jas M. Chok, Omar Kahly, Miranda Miranda, Isabella Jaramillo, Brian Curran, Isabella Santavicca, Lexie Thomas, Maria G Spinelli, Garrett Williams, David K Johnson, David L. Woods

**Affiliations:** ^1^ Neurobehavioral Systems, Inc, Berkeley, CA USA; ^2^ Veterans Affairs Northern California Health Care System, Martinez, CA USA; ^3^ University of Chicago, Chicago, IL USA; ^4^ UC Davis Alzheimer’s Disease Center, Walnut Creek, CA USA

## Abstract

**Background:**

The administration of cognitive tests in patients’ homes facilitates access by underserved communities and increases testing capacity and efficiency. However, the validity of at‐home computerized cognitive tests is often questioned because of limitations in examiner monitoring, distractions, environmental noise, and potential cheating. Here, we compare performance of the computerized and proctored California Cognitive Assessment Battery (CCAB) when administered at‐home or in the laboratory using otherwise identical procedures.

**Method:**

The California Cognitive Assessment Battery (CCAB) includes 17 verbal and 15 non‐verbal tests that have been optimized and normed for at‐home assessment on tablet computers (Figure 1). To reduce the influence of distractions and noise, CCAB verbal stimuli are adjusted for hearing loss and delivered at high intensities through noise‐attenuating circumaural headphones. Verbal responses are digitally recorded using a noise‐cancelling head‐mounted microphone. Most importantly, CCAB test administration is proctored through a control interface that warns of potential error conditions (e.g., performance failures, excessive noise, etc.), displays test performance in real time, facilitates patient observations, and incorporates video chat and test‐control capabilities to readminister tests should problems arise.

During the COVID pandemic 310 participants (mean age 70.1 years) successfully completed three 90‐minute enrollment test sessions in their homes. Omnibus z‐scores were obtained by averaging z‐scores from 70 individual test measures. At 6 months, 277 participants were retested, 46% of them in the laboratory. At 18 months, 200 participants were retested, 57% in the laboratory.

**Result:**

Table 1 shows Omnibus z‐score correlations at enrollment, 6‐, and 18‐ months as a function of retest location: test‐retest reliability was similar for at‐home/in‐lab and at‐home/at‐home pairs. Table 2 shows mean z‐scores for at‐home and in‐lab assessments at 6‐ and 18‐months, estimated with a model that included Omnibus z‐scores at enrollment. No significant differences were observed as a function of test site at 6‐months (*t(273) = ‐0.56, NS)* or 18‐months *(t(172) = 0.44*, *NS*).

**Conclusion:**

The CCAB produces results with similar means, variances, and distributions whether assessments are administered at‐home or in a laboratory.